# 3-(4-Fluoro­phenyl­sulfon­yl)-5-isopropyl-2-methyl-1-benzofuran

**DOI:** 10.1107/S1600536810012869

**Published:** 2010-04-14

**Authors:** Hong Dae Choi, Pil Ja Seo, Byeng Wha Son, Uk Lee

**Affiliations:** a Department of Chemistry, Dongeui University, San 24 Kaya-dong Busanjin-gu, Busan 614-714, Republic of Korea; b Department of Chemistry, Pukyong National University, 599-1 Daeyeon 3-dong, Nam-gu, Busan 608-737, Republic of Korea

## Abstract

In the title compound, C_18_H_17_FO_3_S, the 4-fluoro­phenyl ring makes a dihedral angle of 82.12 (4)° with the plane of the benzofuran fragment. In the crystal structure, mol­ecules are linked by weak inter­molecular C—H⋯O hydrogen bonds and C—H⋯π inter­actions.

## Related literature

For the crystal structures of similar 2-methyl-3-phenyl­sulfonyl-1-benzofuran derivatives, see: Choi *et al.* (2008*a* ▶,*b* ▶). For the biological activity of benzofuran compounds, see: Aslam *et al.* (2006 ▶); Galal *et al.* (2009 ▶); Khan *et al.* (2005 ▶). For natural products with benzofuran rings, see: Akgul & Anil (2003 ▶); Soekamto *et al.* (2003 ▶).
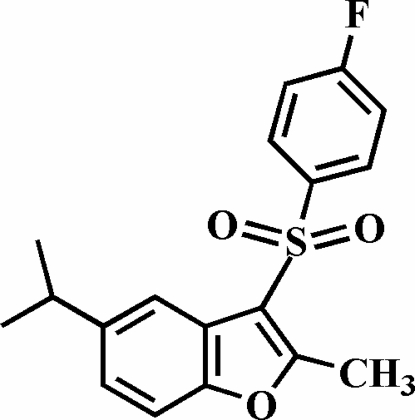


## Experimental

### 

#### Crystal data

C_18_H_17_FO_3_S
*M* *_r_* = 332.38
Monoclinic, 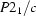

*a* = 10.9434 (6) Å
*b* = 11.3395 (6) Å
*c* = 13.0988 (7) Å
β = 100.252 (3)°
*V* = 1599.51 (15) Å^3^
*Z* = 4
Mo *K*α radiation
μ = 0.23 mm^−1^
*T* = 175 K
0.41 × 0.29 × 0.17 mm

#### Data collection

Bruker SMART APEXII CCD diffractometer
Absorption correction: multi-scan (*SADABS*; Bruker, 2009 ▶) *T*_min_ = 0.669, *T*_max_ = 0.746
14304 measured reflections
3655 independent reflections
3109 reflections with *I* > 2σ(*I*)
*R*_int_ = 0.030

#### Refinement

*R*[*F* ^2^ > 2σ(*F* ^2^)] = 0.037
*wR*(*F* ^2^) = 0.108
*S* = 1.02
3655 reflections
212 parameters
H-atom parameters constrained
Δρ_max_ = 0.36 e Å^−3^
Δρ_min_ = −0.37 e Å^−3^

### 

Data collection: *APEX2* (Bruker, 2009 ▶); cell refinement: *SAINT* (Bruker, 2009 ▶); data reduction: *SAINT*; program(s) used to solve structure: *SHELXS97* (Sheldrick, 2008 ▶); program(s) used to refine structure: *SHELXL97* (Sheldrick, 2008 ▶); molecular graphics: *ORTEP-3* (Farrugia, 1997 ▶) and *DIAMOND* (Brandenburg, 1998 ▶); software used to prepare material for publication: *SHELXL97*.

## Supplementary Material

Crystal structure: contains datablocks global, I. DOI: 10.1107/S1600536810012869/xu2750sup1.cif

Structure factors: contains datablocks I. DOI: 10.1107/S1600536810012869/xu2750Isup2.hkl

Additional supplementary materials:  crystallographic information; 3D view; checkCIF report

## Figures and Tables

**Table 1:** Hydrogen-bond geometry (Å, °) *Cg*1 and *Cg*2 are the centroids of the C1/C2/C7/O3/C8 furan ring and the C2–C7 benzene ring, respectively.

*D*—H⋯*A*	*D*—H	H⋯*A*	*D*⋯*A*	*D*—H⋯*A*
C14—H14⋯O2^i^	0.95	2.51	3.401 (2)	155
C10—H10*B*⋯*Cg*1^ii^	0.98	2.86	3.561 (2)	120
C11—H11*B*⋯*Cg*2^ii^	0.98	2.91	3.670 (2)	135
C17—H17⋯*Cg*2^iii^	0.95	2.89	3.528 (2)	147

Symmetry codes: (i) 
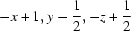
; (ii) 
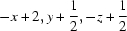
; (iii) 
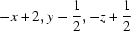
.

**Fig. 1.**
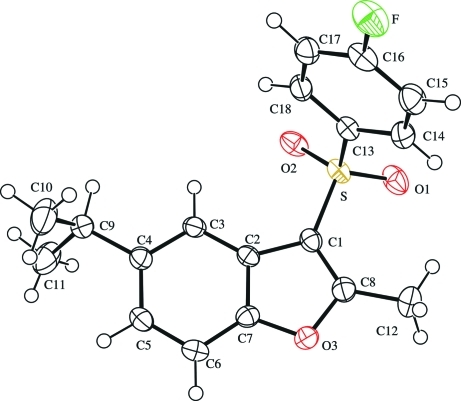
The molecular structure of the title compound with the atom numbering scheme. Displacement ellipsoids are drawn at the 50% probability level. H atoms are presented as a small spheres of arbitrary radius.

**Fig. 2.**
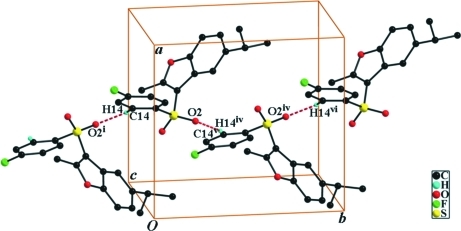
C—H···O interactions (dotted lines) in the crystal structure of the title compound. [Symmetry codes: (i) -x + 1, y - 1/2, -z + 1/2; (iv) -x + 1, y + 1/2, -z + 1/2; (v) x, y + 1, z.]

**Fig. 3.**
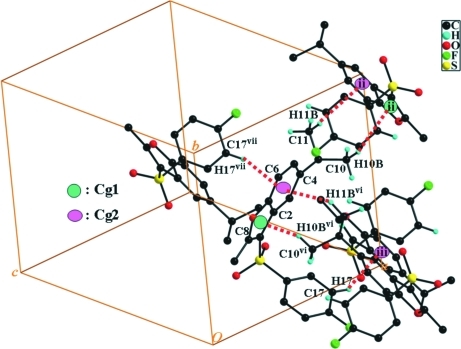
C—H···π interactions (dotted lines) in the crystal structure of the title compound. Cg denotes the ring centroid. [Symmetry codes: (ii) -x + 2, y + 1/2, -z + 1/2; (iii) x, -y + 1/2, z - 1/2; (vi) -x + 2, y - 1/2, -z + 1/2; (vii) x, -y + 1/2, z + 1/2.]

## supplementary crystallographic information

### Comment

The compounds containing benzofuran ring show potent biological activities such as antifungal (Aslam *et al.*, 2006), antitumor and antiviral (Galal *et al.*, 2009), antimicrobial (Khan *et al.*, 2005) properties. These compounds occur widely in nature (Akgul & Anil, 2003; Soekamto *et al.*, 2003). As a part of our ongoing studies of the effect of side chain substituents on the solid state structures of 2-methyl-3-phenylsulfonyl-1-benzofuran analogues (Choi *et al.*, 2008*a*,*b*),  we report the crystal structure of the title compound (Fig. 1).

The benzofuran unit is essentially planar, with a mean deviation of 0.113 (1) Å from the least-squares plane defined by the nine constituent atoms. The 4-fluorophenyl ring makes a dihedral angle of 82.12 (4)° with the plane of the benzofuran fragment. The molecular packing (Fig. 2) is stabilized by an intermolecular C—H···O hydrogen bond between the 4-fluorophenyl H atom and the oxygen of the sulfonyl group, with a C14—H14···O2^i^ (Table 1). The crystal packing (Fig. 3) is further stabilized by three intermolecular C—H···π interactions; the first one between the methyl H atom of the isopropyl group and the furan ring of an adjacent molecule, with a C10—H10B···Cg1^ii^, the second one between the methyl H atom of the isopropyl group and the benzene ring of a neighbouring molecule, with a C11—H11B···Cg2^ii^, and the third one between the 4-fluorophenyl H atom and the benzene ring of an adjacent benzofuran system, with a C17—H17···Cg2^iii^, respectively (Table 1; Cg1 and Cg2 are the centroids of the C1/C2/C7/O3/C8 furan ring and the C2–C7 benzene ring, respectively).

### Experimental

77% 3-Chloroperoxybenzoic acid (448 mg, 2.0 mmol) was added in small portions to a stirred solution of 3-(4-fluorophenylsulfanyl)-5-isoproyl-2-methyl-1-benzofuran (270 mg, 0.9 mmol) in dichloromethane (40 mL) at 273 K. After being stirred at room temperature for 8 h, the mixture was washed with saturated sodium bicarbonate solution and the organic layer was separated, dried over magnesium sulfate, filtered and concentrated at reduced pressure. The residue was purified by column chromatography (silica gel, hexane–ethyl acetate, 2:1 v/v) to afford the title compound as a colorless solid [yield 79%, m.p. 402–403 K; Rf = 0.69 (hexane–ethyl acetate, 2:1 v/v)]. Single crystals suitable for X-ray diffraction were prepared by slow evaporation of a solution of the title compound in benzene at room temperature.

### Refinement

All H atoms were positioned geometrically and refined using a riding model, with C—H = 0.95 Å for aryl, 1.00 Å for methine and 0.98 Å for methyl H atoms, respectively. *U*_iso_(H) = 1.2*U*_eq_(C) for aryl and methine, and 1.5*U*_eq_(C) for methyl H atoms.

### Crystal data

**Table:**

C_18_H_17_FO_3_S	*F*(000) = 696
*M**_r_* = 332.38	*D*_x_ = 1.380 Mg m^−^^3^
Monoclinic, *P*2_1_/*c*	Mo *K*α radiation, λ = 0.71073 Å
Hall symbol: -P 2ybc	Cell parameters from 6367 reflections
*a* = 10.9434 (6) Å	θ = 2.4–27.5°
*b* = 11.3395 (6) Å	µ = 0.23 mm^−^^1^
*c* = 13.0988 (7) Å	*T* = 175 K
β = 100.252 (3)°	Block, colourless
*V* = 1599.51 (15) Å^3^	0.41 × 0.29 × 0.17 mm
*Z* = 4	

### Data collection

**Table:**

Bruker SMART APEXII CCD diffractometer	3655 independent reflections
Radiation source: rotating anode	3109 reflections with *I* > 2σ(*I*)
graphite multilayer	*R*_int_ = 0.030
Detector resolution: 10.0 pixels mm^-1^	θ_max_ = 27.5°, θ_min_ = 2.4°
φ and ω scans	*h* = −14→14
Absorption correction: multi-scan (*SADABS*; Bruker, 2009)	*k* = −14→14
*T*_min_ = 0.669, *T*_max_ = 0.746	*l* = −16→16
14304 measured reflections	

### Refinement

**Table:**

Refinement on *F*^2^	Secondary atom site location: difference Fourier map
Least-squares matrix: full	Hydrogen site location: difference Fourier map
*R*[*F*^2^ > 2σ(*F*^2^)] = 0.037	H-atom parameters constrained
*wR*(*F*^2^) = 0.108	*w* = 1/[σ^2^(*F*_o_^2^) + (0.0594*P*)^2^ + 0.4509*P*] where *P* = (*F*_o_^2^ + 2*F*_c_^2^)/3
*S* = 1.02	(Δ/σ)_max_ < 0.001
3655 reflections	Δρ_max_ = 0.36 e Å^−^^3^
212 parameters	Δρ_min_ = −0.37 e Å^−^^3^
0 restraints	Extinction correction: *SHELXL97* (Sheldrick, 2008), Fc^*^=kFc[1+0.001xFc^2^λ^3^/sin(2θ)]^-1/4^
Primary atom site location: structure-invariant direct methods	Extinction coefficient: 0.0117 (15)

### Special details

**Table:**

Geometry. All esds (except the esd in the dihedral angle between two l.s. planes) are estimated using the full covariance matrix. The cell esds are taken into account individually in the estimation of esds in distances, angles and torsion angles; correlations between esds in cell parameters are only used when they are defined by crystal symmetry. An approximate (isotropic) treatment of cell esds is used for estimating esds involving l.s. planes.
Refinement. Refinement of F^2^ against ALL reflections. The weighted R-factor wR and goodness of fit S are based on F^2^, conventional R-factors R are based on F, with F set to zero for negative F^2^. The threshold expression of F^2^ > 2sigma(F^2^) is used only for calculating R-factors(gt) etc. and is not relevant to the choice of reflections for refinement. R-factors based on F^2^ are statistically about twice as large as those based on F, and R- factors based on ALL data will be even larger.

### Fractional atomic coordinates and isotropic or equivalent isotropic displacement parameters (Å^2^)

**Table:**

	*x*	*y*	*z*	*U*_iso_*/*U*_eq_	
S	0.54221 (3)	0.13196 (3)	0.21925 (3)	0.02922 (13)	
F	0.75485 (9)	−0.21439 (9)	−0.02852 (8)	0.0475 (3)	
O1	0.44396 (10)	0.07695 (11)	0.26153 (9)	0.0415 (3)	
O2	0.51752 (10)	0.23800 (9)	0.15885 (8)	0.0367 (3)	
O3	0.79447 (10)	0.15165 (9)	0.47328 (8)	0.0300 (2)	
C1	0.66335 (13)	0.16383 (12)	0.32124 (11)	0.0268 (3)	
C2	0.75540 (12)	0.25570 (12)	0.32202 (10)	0.0244 (3)	
C3	0.78117 (13)	0.34256 (12)	0.25361 (11)	0.0270 (3)	
H3	0.7288	0.3527	0.1881	0.032*	
C4	0.88480 (13)	0.41423 (13)	0.28261 (11)	0.0285 (3)	
C5	0.96004 (13)	0.39900 (13)	0.38020 (11)	0.0302 (3)	
H5	1.0300	0.4491	0.3993	0.036*	
C6	0.93628 (13)	0.31367 (13)	0.44994 (11)	0.0297 (3)	
H6	0.9877	0.3038	0.5159	0.036*	
C7	0.83340 (13)	0.24398 (12)	0.41752 (10)	0.0257 (3)	
C8	0.69246 (14)	0.10323 (13)	0.41204 (11)	0.0303 (3)	
C9	0.91774 (15)	0.50549 (14)	0.20723 (12)	0.0356 (4)	
H9	0.8483	0.5073	0.1462	0.043*	
C10	1.0351 (2)	0.47032 (18)	0.16761 (16)	0.0548 (5)	
H10A	1.1061	0.4728	0.2249	0.082*	
H10B	1.0492	0.5253	0.1132	0.082*	
H10C	1.0256	0.3902	0.1392	0.082*	
C11	0.9299 (2)	0.62926 (15)	0.25374 (16)	0.0531 (5)	
H11A	0.8558	0.6477	0.2832	0.080*	
H11B	0.9381	0.6866	0.1994	0.080*	
H11C	1.0036	0.6329	0.3085	0.080*	
C12	0.64435 (18)	−0.00464 (15)	0.45463 (14)	0.0452 (4)	
H12A	0.5675	−0.0294	0.4094	0.068*	
H12B	0.6275	0.0117	0.5243	0.068*	
H12C	0.7062	−0.0677	0.4584	0.068*	
C13	0.60386 (13)	0.02725 (12)	0.14291 (11)	0.0274 (3)	
C14	0.60321 (15)	−0.09153 (13)	0.16991 (12)	0.0342 (3)	
H14	0.5684	−0.1159	0.2279	0.041*	
C15	0.65382 (15)	−0.17364 (14)	0.11133 (13)	0.0375 (4)	
H15	0.6546	−0.2551	0.1283	0.045*	
C16	0.70315 (14)	−0.13427 (13)	0.02763 (13)	0.0332 (3)	
C17	0.70304 (14)	−0.01834 (14)	−0.00155 (12)	0.0344 (3)	
H17	0.7364	0.0049	−0.0606	0.041*	
C18	0.65300 (14)	0.06423 (13)	0.05734 (12)	0.0309 (3)	
H18	0.6523	0.1454	0.0394	0.037*	

### Atomic displacement parameters (Å^2^)

**Table:**

	*U*^11^	*U*^22^	*U*^33^	*U*^12^	*U*^13^	*U*^23^
S	0.0237 (2)	0.0283 (2)	0.0349 (2)	−0.00195 (13)	0.00319 (14)	−0.00717 (14)
F	0.0421 (6)	0.0428 (6)	0.0595 (7)	0.0017 (4)	0.0145 (5)	−0.0195 (5)
O1	0.0295 (6)	0.0473 (7)	0.0503 (7)	−0.0098 (5)	0.0138 (5)	−0.0133 (5)
O2	0.0336 (6)	0.0308 (6)	0.0412 (6)	0.0052 (4)	−0.0057 (5)	−0.0056 (5)
O3	0.0344 (6)	0.0300 (5)	0.0254 (5)	−0.0011 (4)	0.0047 (4)	0.0015 (4)
C1	0.0263 (7)	0.0248 (7)	0.0296 (7)	−0.0011 (5)	0.0061 (5)	−0.0042 (5)
C2	0.0238 (6)	0.0237 (7)	0.0254 (7)	0.0010 (5)	0.0033 (5)	−0.0044 (5)
C3	0.0282 (7)	0.0272 (7)	0.0238 (7)	−0.0006 (5)	0.0001 (5)	−0.0015 (5)
C4	0.0309 (7)	0.0270 (7)	0.0280 (7)	−0.0026 (6)	0.0060 (6)	−0.0031 (6)
C5	0.0276 (7)	0.0305 (7)	0.0317 (8)	−0.0043 (6)	0.0032 (6)	−0.0058 (6)
C6	0.0299 (7)	0.0317 (7)	0.0253 (7)	0.0021 (6)	−0.0009 (5)	−0.0044 (6)
C7	0.0296 (7)	0.0248 (7)	0.0229 (7)	0.0026 (5)	0.0055 (5)	−0.0014 (5)
C8	0.0318 (7)	0.0298 (7)	0.0307 (8)	−0.0016 (6)	0.0096 (6)	−0.0033 (6)
C9	0.0429 (9)	0.0341 (8)	0.0288 (7)	−0.0112 (7)	0.0035 (6)	0.0009 (6)
C10	0.0683 (13)	0.0476 (11)	0.0569 (12)	−0.0131 (9)	0.0341 (10)	−0.0023 (9)
C11	0.0834 (14)	0.0304 (9)	0.0503 (11)	−0.0054 (9)	0.0247 (10)	0.0037 (8)
C12	0.0516 (10)	0.0413 (9)	0.0444 (10)	−0.0111 (8)	0.0130 (8)	0.0077 (8)
C13	0.0245 (7)	0.0263 (7)	0.0302 (7)	−0.0030 (5)	0.0023 (5)	−0.0041 (6)
C14	0.0398 (8)	0.0281 (7)	0.0351 (8)	−0.0050 (6)	0.0080 (6)	−0.0006 (6)
C15	0.0423 (9)	0.0249 (7)	0.0445 (9)	−0.0006 (6)	0.0054 (7)	−0.0022 (7)
C16	0.0248 (7)	0.0340 (8)	0.0396 (9)	−0.0004 (6)	0.0029 (6)	−0.0126 (6)
C17	0.0315 (8)	0.0378 (8)	0.0349 (8)	−0.0083 (6)	0.0087 (6)	−0.0040 (6)
C18	0.0303 (7)	0.0269 (7)	0.0350 (8)	−0.0057 (6)	0.0046 (6)	−0.0004 (6)

### Geometric parameters (Å, °)

**Table:**

S—O1	1.437 (1)	C9—C11	1.526 (2)
S—O2	1.438 (1)	C9—H9	1.0000
S—C1	1.744 (2)	C10—H10A	0.9800
S—C13	1.762 (1)	C10—H10B	0.9800
F—C16	1.355 (2)	C10—H10C	0.9800
O3—C8	1.368 (2)	C11—H11A	0.9800
O3—C7	1.386 (2)	C11—H11B	0.9800
C1—C8	1.361 (2)	C11—H11C	0.9800
C1—C2	1.448 (2)	C12—H12A	0.9800
C2—C7	1.390 (2)	C12—H12B	0.9800
C2—C3	1.394 (2)	C12—H12C	0.9800
C3—C4	1.392 (2)	C13—C14	1.393 (2)
C3—H3	0.9500	C13—C18	1.391 (2)
C4—C5	1.403 (2)	C14—C15	1.383 (2)
C4—C9	1.517 (2)	C14—H14	0.9500
C5—C6	1.387 (2)	C15—C16	1.380 (2)
C5—H5	0.9500	C15—H15	0.9500
C6—C7	1.379 (2)	C16—C17	1.369 (2)
C6—H6	0.9500	C17—C18	1.386 (2)
C8—C12	1.480 (2)	C17—H17	0.9500
C9—C10	1.521 (2)	C18—H18	0.9500
			
O1—S—O2	119.66 (7)	C9—C10—H10A	109.5
O1—S—C1	108.28 (7)	C9—C10—H10B	109.5
O2—S—C1	106.91 (7)	H10A—C10—H10B	109.5
O1—S—C13	108.33 (7)	C9—C10—H10C	109.5
O2—S—C13	107.74 (7)	H10A—C10—H10C	109.5
C1—S—C13	104.99 (7)	H10B—C10—H10C	109.5
C8—O3—C7	106.82 (11)	C9—C11—H11A	109.5
C8—C1—C2	107.62 (13)	C9—C11—H11B	109.5
C8—C1—S	126.33 (11)	H11A—C11—H11B	109.5
C2—C1—S	125.97 (11)	C9—C11—H11C	109.5
C7—C2—C3	118.96 (12)	H11A—C11—H11C	109.5
C7—C2—C1	104.63 (12)	H11B—C11—H11C	109.5
C3—C2—C1	136.39 (13)	C8—C12—H12A	109.5
C4—C3—C2	119.04 (13)	C8—C12—H12B	109.5
C4—C3—H3	120.5	H12A—C12—H12B	109.5
C2—C3—H3	120.5	C8—C12—H12C	109.5
C3—C4—C5	119.64 (13)	H12A—C12—H12C	109.5
C3—C4—C9	119.80 (13)	H12B—C12—H12C	109.5
C5—C4—C9	120.53 (13)	C14—C13—C18	121.08 (14)
C6—C5—C4	122.53 (13)	C14—C13—S	119.19 (11)
C6—C5—H5	118.7	C18—C13—S	119.73 (11)
C4—C5—H5	118.7	C15—C14—C13	119.29 (14)
C7—C6—C5	115.82 (13)	C15—C14—H14	120.4
C7—C6—H6	122.1	C13—C14—H14	120.4
C5—C6—H6	122.1	C16—C15—C14	118.36 (14)
C6—C7—O3	125.53 (13)	C16—C15—H15	120.8
C6—C7—C2	124.01 (13)	C14—C15—H15	120.8
O3—C7—C2	110.45 (12)	F—C16—C17	118.17 (14)
C1—C8—O3	110.44 (13)	F—C16—C15	118.35 (14)
C1—C8—C12	134.67 (15)	C17—C16—C15	123.48 (14)
O3—C8—C12	114.75 (13)	C16—C17—C18	118.29 (14)
C4—C9—C10	110.85 (14)	C16—C17—H17	120.9
C4—C9—C11	112.42 (13)	C18—C17—H17	120.9
C10—C9—C11	110.99 (15)	C17—C18—C13	119.49 (14)
C4—C9—H9	107.4	C17—C18—H18	120.3
C10—C9—H9	107.4	C13—C18—H18	120.3
C11—C9—H9	107.4		

### Hydrogen-bond geometry (Å, °)

**Table:**

*D*—H···*A*	*D*—H	H···*A*	*D*···*A*	*D*—H···*A*
C14—H14···O2^i^	0.95	2.51	3.401 (2)	155
C10—H10B···Cg1^ii^	0.98	2.86	3.561 (2)	120
C11—H11B···Cg2^ii^	0.98	2.91	3.670 (2)	135
C17—H17···Cg2^iii^	0.95	2.89	3.528 (2)	147

Symmetry codes: (i) −*x*+1, *y*−1/2, −*z*+1/2; (ii) −*x*+2, *y*+1/2, −*z*+1/2; (iii) −*x*+2, *y*−1/2, −*z*+1/2.
